# Patterns of participation over four rounds of annual fecal immunochemical test-based screening for colorectal cancer: what predicts rescreening?

**DOI:** 10.1186/s12889-017-4634-8

**Published:** 2017-08-01

**Authors:** Joanne M. Osborne, Carlene Wilson, Amy Duncan, Stephen R. Cole, Ingrid Flight, Deborah Turnbull, Donna L. Hughes, Graeme P. Young

**Affiliations:** 1 Bowel Health Service and Flinders Centre for Innovation in Cancer, Adelaide, Australia; 2 University of Adelaide, North Terrace, Adelaide, SA 5005 Australia; 3 Flinders Centre for Innovation in Cancer, Flinders University of South Australia, GPO Box 2100, Adelaide, SA 5001 Australia; 4 Olivia Newton John Cancer, Wellness and Research Centre, Heidelberg, Victoria 3084 Australia; 5 School of Psychology and Public Health, La Trobe University, Bundoora, Victoria 3086 Australia

**Keywords:** Colorectal cancer, Screening, Re-screening, Fecal occult blood test, Adherence, Dissatisfaction

## Abstract

**Background:**

Participation at the recommended intervals is critical for screening to be effective in reducing colorectal cancer (CRC) incidence. This study describes patterns of screening participation over four rounds of fecal immunochemical testing (FIT) to identify whether demographic variables and prior screening satisfaction are significantly associated with patterns of re-participation.

**Methods:**

Baseline surveys were mailed to 4000 South Australians randomly selected from the electoral-roll. Respondents (*n* = 1928/48.2%) were offered four annual FIT rounds. Screening participation and satisfaction at each round were recorded.

**Results:**

Study participation was 58.5, 66.9, 73.1 and 71.4% respectively over four rounds. Three participation patterns were described: consistent participation (43.1%), consistent non-participation (26.4%) and inconsistent participation (changeable; 30.5%), including intermittent and sustained change patterns. Sustained change described those who changed participatory behavior and then maintained for at least two rounds (*n* = 375/19.5%). Older people, and those not working were most likely to sustain participation. Younger invitees, especially men, were more likely to change participatory behavior and sustain the change. People with higher disadvantage, less education, not working and with no prior (pre-trial) screening experience were more likely to start participating and drop out. People dissatisfied with a prior screening test, including finding aspects embarrassing or unpleasant, were also more likely not to participate in annual screening or to drop out.

**Conclusions:**

The findings identify those at risk of non- or inconsistent participation in rescreening. They should aid targeting of interventions for demographic groups at risk and ensuring screening experiences are not perceived as unpleasant or difficult.

## Background

Participation at recommended intervals in fecal-occult blood test-(FOBT)-based population screening for colorectal cancer (CRC), by either guaiac (gFOBT) or immunochemical FOBT (i.e., fecal immunochemical test [FIT]) is associated with decreased population mortality [1–3]. For screening to work effectively to reduce population CRC burden, participation patterns should conform to evidence-based screening intervals. In Australia, guidelines recommend an interval of at least once every 2 years for average-risk people aged 50–75 [4] whereas the USA recommendation is annually after turning 50 [5]. The evidence base supports annual screening [1].

Despite the evidence, participation and re-participation rates are sub-optimal in Australia and many other countries [6, 7]. Identifying demographic and other variables associated with participation, and particularly consistency in re-participation, would enable the identification of population subgroups that might benefit from additional support to screen, or events that might trigger withdrawal from re-screening, and help in the development of targeted interventions to improve outcomes.

Different cancer screening participation patterns have been described [8]: people who adhere to all screening offers (consistent participants); never participate (consistent non-participants); or participate inconsistently (intermittent/changeable). The latter includes those who respond to subsequent offers after rejecting the first (late entrants), those who participate initially but then “drop-out” and those who screen sporadically. Testing whether these different patterns of inconsistency are behaviorally meaningful (i.e., reflect an underlying difference in attitude to, or perception of, screening) is important to assist in identifying strategies to optimize CRC prevention.

Some limited research has been undertaken investigating participants in cancer screening who “drop-out” of programs. This group has been contrasted with consistent participants and non-participants because they represent people who make a behavioral choice that is not sustained but may be amenable to change. For example, a study investigating participatory behavior over two rounds of mammography screening [9] found that women aged below 50 years who self-reported having a limiting long term illness were significantly more likely to be inconsistent screeners. In previous studies investigating CRC rescreening over three rounds, inconsistent screening was associated with a negative prior screening experience [10, 11], as well as being younger, male, having reduced self-efficacy for screening, and lower perceived health practitioner support [11]. Non-participation in screening has also been associated with test type and fecal aversion [11–14]. In one study [14], normalising the fecal testing process by discussing it in a social setting led to subsequent participation in screening (‘late entrant’ participation). However, an analysis of only three rounds of participation is not sufficient to ascertain if changes are sustained over future rounds.

The extent of consistency in participation that represents a meaningful commitment to screening for CRC also remains to be determined. Clearly, there may be short-term reasons for non-participation; acute ill-health, postage failure, travel, or significant life challenges. The clearest indication of a problematic pattern of re-participation is where a previously consistent screener becomes a consistent non-screener. Understanding such a sustained change might identify previously unknown barriers. Conversely, lessons can also be learned from success; identifying reasons for sustained change from previous non-participation to re-participation might provide information as to how to engage people in screening and sustain optimal behavior.

We aim to extend Lo et al. [10] and our own earlier findings [11] by exploring patterns of participation across four annual rounds of FIT screening provided to an Australian sample of the general population, and to identify factors associated with participation over time.

## Methods

### Study population

As described elsewhere [11], a random sample of 4000 South Australian men and women aged 50–74 was drawn from the Australian Electoral Roll.

### Study design

The study consisted of an initial Baseline survey and a subsequent screening phase (offered to those who completed the Baseline survey) conducted annually for 4 years. The Baseline survey is described elsewhere [15].

Round 1 screening invitations were mailed to those who completed the Baseline survey from the Bowel Health Service at Repatriation General Hospital Daw Park between November 2008 and February 2009. Invitees received a screening invitation letter, 2 OC-Sensor sample tubes (Eiken Chemical Co., Japan), an instruction brochure, a participant details and consent form, a screening status form, a short survey regarding aspects of their screening experience, and a reply-paid envelope.

Subsequently, similar invitations were mailed in the last quarter of each year 2009–2011. With each invitation, participants received a survey to record screening experiences and satisfaction with the screening process. General satisfaction was measured with two items (satisfaction with the screening service and satisfaction with their decision) using a five-point Likert scale (1 = Very Satisfied to 5 = Very Unsatisfied). Participants also rated their positive (worthwhile, convenient, reassuring, easy to complete) and negative (embarrassing, unpleasant) screening experiences on a Likert scale (1 = Strongly Agree to 5 = Strongly Disagree).

All survey participants were mailed the four screening invitations irrespective of participation in prior screening rounds unless excluded from future FIT offers because they requested to opt out, or because they reported that they had completed investigations that precluded the need for further screening offers.

Participation in any round was defined as returning the two fecal samples from that round’s offer at any time between the day after they were sent the screening offer and kit until the day they were sent the next offer and kit. Participation was coded as Yes or No. After four screening offers, 16 different behavioral response patterns were possible. These will be described in the Results section.

### Analyses

Screening participation per round and longitudinally over rounds were described via frequency distributions, and Chi-Square analyses examined univariate differences between invitees in different participation categories. Differences between reported positive and negative experiences and subsequent screening behavior were investigated with t-test analyses. Binary logistic regression was used to determine whether initial or recent experiences had more impact on subsequent screening. Statistical Package for the Social Sciences (SPSS,v.19) was used.

## Results

### Survey-respondent characteristics

Baseline surveys were returned by 1928/4000 respondents (48.2%). Survey-respondents were 52.5% female (*n* = 1013), aged 50–75 (*M* = 60.32, *SD* = 6.60), and most were married (*n* = 1487, 77%). About half (50.7%, *n* = 953) were currently in the workforce (full or part-time) and unemployment was low (*n* = 56, 3%); other participants were retired or home carers. Most reported at least secondary school completion (*n* = 1238, 64.2%), and 50.2% of these had completed higher education. The majority (*n* = 1349, 70.0%) were born in Australia and spoke English at home (*n* = 1628, 84.4%); for others, the mean number of years in Australia was 40.69 (*SD* = 12.21). Only 20.5% (*n* = 395) reported no private health insurance, 18.2% (*n* = 350) reported a family history of CRC, which is slightly higher than the familial population rate of 10–15% reported by Kerber and colleagues in 2005 [16].

Measuring relative socio-economic disadvantage based on post-code [17] showed low levels of disadvantage among participants with the index ranging from 756 to 1124 (*M* = 997.98,*SD* = 68.017), a range representative of 97% of the wider South Australian population as determined by the 2006 Census [18].

### Participation in each of the four rounds

**Fig. 1:**
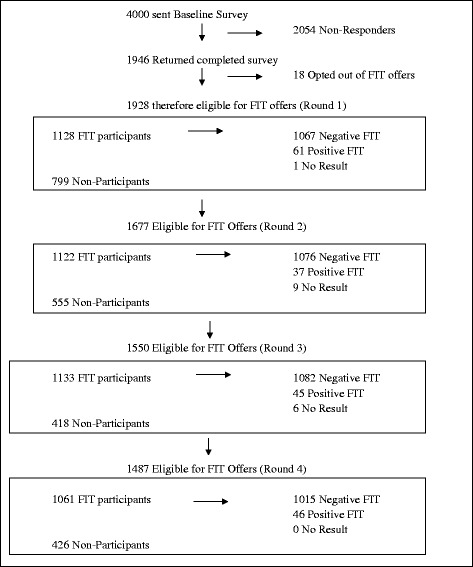
Study participation flow diagram over four rounds of screening

Figure 1 is a Consort diagram showing the four-round screening pathway with study attrition. Participation at each round was 58.6% (1128/1928), 66.9% (1122/1677), 73% (1133/1550) and 71.4% (1061/1487) respectively. Most survey-respondents returned kits in at least one round (*n* = 1445, 74.9%); 1217 (63.1%) participated in at least two rounds, 1014 (52.6%) participated in at least three and 762 (39.5%) participated in all four rounds.

**Fig. 2:**
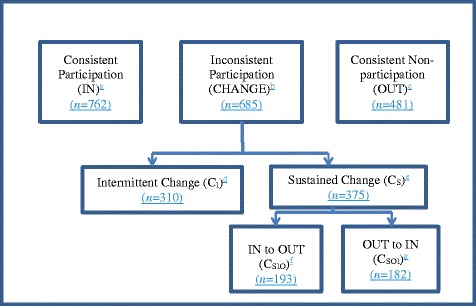
Participatory behavior category flow chart. Participation patterns: ^a^YYYY; ^b^YNNN, NYYY, YYNN, NNYY, NYNN, YNYY, NNYN, YYNY, NYYN, YNNY, NYNY, YNYN, YYYN, NNNY; ^c^NNNN; ^d^NYNN, YNYY, NNYN, YYNY,NYNY, YNYN, NYYN, YNNY, YYYN, NNNY; ^e^YNNN, NYYY, YYNN, NNYY; ^f^YNNN, YYNN; ^g^NYYY, NNYY

Nearly 40 % (39.5%: 762/1928) of the survey-respondents participated in all four annual rounds (IN). A further 35.5% (685/1928) had an inconsistent participation pattern (CHANGE), and the remaining 24.9% (481/1928) did not participate in any of the four rounds (OUT). Figure 2 depicts the possible participatory behavior patterns. The number of participants in each classification category is also shown in Fig. 2. Each classification category is named for easy future reference, and participation patterns in each category described.

### Demographic associations with participation patterns

Table 1 presents the significant results of chi-square analyses. Comparisons between those who always participated (IN) and those who never did (OUT), showed IN respondents were more likely to be female, older (55–74), not working, and with prior (pre-study) FIT experience. When comparing IN with those who changed participation (CHANGE), the IN respondents were more likely to be married and also more likely to be older (55–74), not working and with prior FOBT experience. Comparing the CHANGE participants with those who never participated (OUT) showed the latter to be more likely to have no prior FOBT experience.

**Table 1:** Significant univariate differences in demographics between different participatory categorisations

Demographic	Study FIT Participation Pattern n (%)			ChiSquare
	Total: IN & OUT	Sustained Participation (IN)	Sustained Non-Participation (OUT)	
Gender				
Males	579 (46.6)	338 (44.4)	241 (50.1)	χ^2^(1) = 3.686, *p* = .055^a^
Females	664 (53.4)	424 (55.6)	240 (49.9)	
Total	1243 (100)	762 (100)	481 (100)	
Age				
50–54	292 (23.5)	128 (16.8)	164 (34.1)	χ^2^(2) = 55.081, *p* < .001**
55–64	607 (48.8)	387 (50.8)	220 (45.7)	
65–74	344 (27.7)	247 (32.4)	97 (20.2)	
Total	1243 (100)	762 (100)	481 (100)	
Employment Status				
Not in Workforce	622 (51.2)	409 (54.8)	213 (45.4)	χ^2^(1) = 9.681, *p* = .002*
In Workforce	594 (48.8)	338 (45.2)	256 (54.6)	
Total	1216 (100)	747 (100)	469 (100)	
Baseline Survey				
No prior FOBT experience	622 (51.0)	342 (45.6)	280 (59.6)	χ^2^(1) = 22.023, *p* < .001**
Prior FOBT experience	598 (49.0)	408 (54.4)	190 (40.4)	
Total	1220 (100)	750 (100)	470 (100)	
	Total: IN & CHANGE	Sustained Participation (IN)	Changeable (CHANGE)	
Marital Status				
Unmarried	308 (21.5)	147 (19.4)	161 (23.8)	χ^2^(1) = 3.728, *p* = .053^a^
Married/DeFacto	1125 (78.5)	609 (80.6)	516 (76.2)	
Total	1433 (100)	756 (100)	677 (100)	
Age				
50–54	321 (22.2)	128 (16.8)	193 (28.2)	χ^2^(2) = 30.255, *p* < .001**
55–64	713 (49.3)	387 (50.8)	326 (47.6)	
65–74	413 (28.5)	247 (32.4)	166 (24.2)	
Total	1447 (100)	762 (100)	685 (100)	
Employment Status				
Not in Workforce	714 (50.6)	409 (54.8)	305 (45.9)	χ^2^(1) = 10.587, *p* = .001**
In Workforce	697 (49.4)	338 (45.2)	359 (54.1)	
Total	1411 (100)	747 (100)	664 (100)	
Baseline Survey				
No prior FOBT experience	687 (48.4)	342 (45.6)	345 (51.6)	χ^2^(1) = 4.809, *p* = .028*
Prior FOBT experience	732 (51.6)	408 (54.4)	324 (48.4)	
Total	1419 (100)	750 (100)	669 (100)	
	Total: CHANGE & OUT	Changeable (CHANGE)	Sustained Non-Participation (OUT)	
Baseline Survey				
No prior FOBT experience	625 (54.9)	345 (51.6)	280 (59.6)	χ^2^(1) = 6.824, *p* = 0.009*
Prior FOBT experience	514 (45.1)	324 (48.4)	190 (40.4)	
Total	1139 (100)	669 (100)	470 (100)	
	Total: IN & C_S_	Sustained Participation (IN)	Sustained Change (C_S_)	
Gender				
Males	538 (47.3)	338 (44.4)	200 (53.3)	χ^2^(1) = 7.767, *p* = .005*
Females	599 (52.7)	424 (55.6)	175 (46.7)	
Total	1137 (100)	762 (100)	375 (100)	
Age				
50–54	232 (20.4)	128 (16.8)	104 (27.7)	χ^2^(2) = 18.526, *p* < 001**
55–64	551 (48.5)	387 (50.8)	164 (43.7)	
65–74	354 (31.1)	247 (32.4)	107 (28.5)	
Total	1137 (100)	762 (100)	375 (100)	
	Total: OUT & C_S_	Sustained Non-Participation (OUT)	Sustained Change (C_S_)	
Age				
50–54	268 (31.3)	164 (34.1)	104 (27.7)	χ^2^(2) = 9.103, *p* = .011*
55–64	384 (44.9)	220 (45.7)	164 (43.7)	
65–74	204 (23.8)	97 (20.2)	107 (28.5)	
Total	856 (100)	481 (100)	375 (100)	
Baseline Survey				
No prior FOBT experience	460 (55.2)	280 (59.6)	180 (49.5)	χ^2^(1) = 8.096, *p* = .004*
Prior FOBT experience	374 (44.8)	190 (40.4)	184 (50.5)	
Total	834 (100)	470 (100)	364 (100)	
	Total: C_I_ & C_S_	Intermittent (C_I_)	Sustained Change (C_S_)	
Gender				
Males	336 (49.1)	136 (43.9)	200 (53.3)	χ^2^(1) = 5.707, *p* = .017*
Females	349 (50.9)	174 (56.1)	175 (46.7)	
Total	685 (100)	310 (100)	375 (100)	
Age				
50–54	193 (28.2)	89 (28.7)	104 (27.7)	χ^2^(2) = 8.970, *p* = .011*
55–64	326 (47.6)	162 (52.3)	164 (43.7)	
65–74	166 (24.2)	59 (19.0)	107 (28.5)	
Total	685 (100)	310 (100)	375 (100)	
	Total: IN & C_SIO_	Sustained Participation (IN)	Sustained Change (in to out) (C_SIO_)	
Gender				
Males	440 (46.1)	338 (44.4)	102 (52.8)	χ^2^(1) = 4.135, *p* = .042*
Females	515 (53.9)	424 (55.6)	91 (47.2)	
	955 (100)	762 (100)	193 (100)	
Age				
50–54	182 (19.1)	128 (16.8)	54 (28.0)	χ^2^(2) = 14.240, *p* < .001**
55–64	463 (48.5)	387 (50.8)	76 (39.4)	
65–74	310 (32.5)	247 (32.4)	63 (32.6)	
Total	955 (100)	762 (100)	193 (100)	
Tertile of Economic Disadvantage				
High Disadvantage	206 (21.6)	156 (20.5)	50 (25.9)	χ^2^(2) = 8.432, *p* = .015*
Medium Disadvantage	329 (34.5)	253 (33.2)	76 (39.4)	
Low Disadvantage	419 (43.9)	352 (46.3)	67 (34.7)	
Total	954 (100)	761 (100)	262 (100)	
Baseline Survey				
No prior FOBT experience	454 (48.5)	342 (45.6)	112 (60.2)	χ^2^(1) = 12.167, *p* < .001**
Prior FOBT experience	482 (51.5)	408 (54.4)	74 (39.8)	
Total	936 (100)	750 (100)	186 (100)	
	Total: C_SOI_ & OUT	Sustained Change (out to in) (C_SOI)_	Sustained Non-Participation (OUT)	
Baseline Survey				
No prior FOBT experience	348 (53.7)	68 (38.2)	280 (59.6)	χ^2^(1) = 22.8671, *p* < .001**
Prior FOBT experience	300 (46.3)	110 (61.8)	190 (40.4)	
Total	648(100)	178 (100)	470 (100)	
	Total: C_SIO_ & C_SOI_	Sustained Change (in to out) (C_SIO_)	Sustained Change (out to in) (C_SOI_)	
Employment Status				
Not in Workforce	179 (49.0)	106 (56.7)	73 (41.0)	χ^2^(1) = 8.348, *p* = .004*
In Workforce	186 (51.0)	81 (43.3)	105 (59.0)	
Total	365 (100)	187 (100)	178 (100)	
Tertile of Economic Disadvantage				
High Disadvantage	86 (22.9)	50 (25.9)	36 (19.8)	χ^2^(2) = 9.619, *p* = .008*
Medium Disadvantage	130 (34.7)	76 (39.4)	54 (29.7)	
Low Disadvantage	159 (42.4)	67 (34.7)	92 (50.5)	
Total	375 (100)	193 (100)	182 (100)	
Education Status				
Lower education (completed high school or lower)	192 (52.6)	110 (59.1)	82 (52.6)	χ^2^(1) = 5.977, *p* = .014*
Higher education (>high school)	173 (47.4)	76 (40.9)	97 (54.2)	
Total	365 (100)	186 (100)	179 (100)	
Baseline Survey				
No prior FOBT experience	180 (49.5)	112 (60.2)	68 (38.2)	χ^2^(1) = 16.762, *p* < .001**
Prior FOBT experience	184 (50.5)	74 (39.8)	110 (61.8)	
Total	364 (100)	186 (100)	178 (100)	

*p < .05; **p < .001

^a^trend towards significance

Closer examination of the CHANGE group (comparing subsets of participatory behavior within the CHANGE group) showed that, where change in participation was sustained (C_S_, *n* = 375) rather than intermittent (C_I_, *n* = 310), the C_S_ group was older (55–74), and male. Further exploration of the sustained change (Cs) participation pattern was done by comparing this group to those with a seemingly committed position, IN or OUT. When compared to those in the IN pattern, those who changed participation and sustained it (Cs) were more likely to be younger and male. When compared to the OUT group, the Cs group were more likely to be older and have some prior (pre-trial) FIT experience. Comparing consistently IN with C_SIO_ (those in the Sustained Change category who changed from In to Out at any point after the first offer) revealed that those who dropped out were more likely to be male, younger (50–54), with higher economic disadvantage and no prior FIT experience. Conversely, when comparing consistently OUT with C_SOI_ (those in the Sustained Change category who changed from Out to In at any point after the first offer,), the latter were significantly more likely to have prior FIT experience.

Finally, comparing the Sustained Change categories C_SIO_ (those who dropped out of annual screening) with C_SOI_ (those who commenced participation after round 1), the ‘late entrants’ (C_SOI_) were more likely to be in the workforce, have lower economic disadvantage, higher education, and prior FIT experience than the ‘drop outs’ (C_SIO_). From these analyses, it appears that prior experience with FIT is a key factor in determining whether someone will respond to a re-screening offer.

### Prior FIT experience and satisfaction

Prior (pre-trial) FIT experience was explored further to discern how it influenced the rescreening decision. Almost half of the respondents (47.8%: 922/1928) reported FIT experience prior to the study. Almost all of these (96.6%: 891/922) completed the optional baseline survey question measuring satisfaction with prior (pre-study) screening. Of those who completed the question, 85.7% (764/891) were either satisfied or very satisfied with the experience. Comparisons between participation categories IN and OUT, between IN and CHANGE and between IN and those who changed from In to Out and sustained this change (C_SIO_), revealed significant differences related to satisfaction with prior screening and report of negative screening experiences, with those who were more satisfied with prior FIT experience and who rated their past experience as less embarrassing and unpleasant more likely to sustain participation (IN) (see Table 2).

**Table 2:** Significant t-test comparisons of impact of prior FOBT experience and satisfaction on subsequent screening behavior patterns

Experience variable	Behavior group	Frequency (n)	Mean	t-test result
Sustained In (IN) and Sustained Out (OUT)				
Prior FOBT satisfaction	IN	408	4.38	*t* (306) = 2.07, *p* = .040*
	OUT	190	4.22	
Sustained In (IN) and Change (CHANGE)				
Prior FOBT satisfaction	IN	408	4.38	*t* (718) = 3.59, *p* < 001**
	CHANGE	312	4.16	
Round 1 Negative^b^	IN	611	4.55	*t* (885) = −2.69, *p* = .007*
	CHANGE	276	4.93	
Sustained In (IN) and Sustained Change In to Out(C_SIO_)				
Prior FOBT satisfaction	IN	408	4.38	*t* (478) = 2.38, *p* = .018*
	C_SIO_	72	4.14	
Round 1 Negative^b^	IN	611	4.55	*t* (761) = −2.72, *p* = .007*
	C_SIO_	152	5.03	
Prior experience and Round 2 participation				
Prior FOBT satisfaction	Rnd 2 participant	583	4.30	*t* (908) = 1.72, *p* = .085 ^a^
	Rnd 2 non-participant	327	4.20	
Round 1 Negative^b^	Rnd 2 participant	728	4.56	*t* (885) = −3.54, *p* < 001**
	Rnd 2 non-participant	159	5.16	
Prior experience and Round 3 participation				
Prior FOBT satisfaction	Rnd 3 participant	597	4.33	*t* (908) = 3.03, *p* = .005*
	Rnd 3 non-participant	313	4.15	
Round 1 Negative^b^	Rnd 3 participant	708	4.57	*t* (885) = −3.06, *p* = .002*
	Rnd 3 non-participant	179	5.07	
Round 2 Negative^b^	Rnd 3 participant	766	4.22	*t* (856) = −2.85, *p* = .004*
	Rnd 3 non-participant	92	4.83	
Round 2 General satisfaction	Rnd 3 participant	766	9.16	*t* (856) = 2.83, *p* = .005*
	Rnd 3 non-participant	92	8.76	
Prior experience and Round 4 participation				
Prior FOBT satisfaction	Rnd 4 participant	562	4.33	*t* (908) = 2.66, *p* = .008*
	Rnd 4 non-participant	348	4.18	
Round 3 General satisfaction	Rnd 4 participant	906	4.02	*t* (1027) = −2.42, *p* = .016*
	Rnd 4 non-participant	123	4.39	

^a^trend towards significance

^b^negative experiences (summary score of negative experiences – embarrassing and unpleasant)

*p < .05; **p < .001

Associations between prior (pre-study) FIT satisfaction and experiences and participation in subsequent rounds were also explored (see Table 2). Satisfaction with pre-study FIT screening from any source was significantly associated with subsequent participation; participants at each round reported higher prior FIT satisfaction compared to non-participants. In addition, non-participation in either Rounds 2 or 3 was associated with negative experiences (embarrassment and unpleasantness) reported at the previous screening round within the study. Being generally satisfied with screening Round 2 was associated with participation in Round 3. Conversely, Round 4 non-participants rated their general satisfaction in Round 3 higher than participants.

Binary Logistic Regression analyses were undertaken to examine whether behavior (participation/non-participation) at Round 3 was predicted by the most recent or less recent FIT testing experience. The analysis was framed using a 2 block-entry method including the significant factors identified in the t-test analysis. The first block consisted of Round 1 negative experiences, and block 2 consisted of Round 2 negative experiences and Round 2 general satisfaction. Inclusion required completion of survey experience questions from both Round 1 and Round 2. This reduced the sample size to 536 participants and 47 non-participants. The outcome was non-participation in Round 3. All predictors analysed were non-significant. These results suggest that negative prior FIT experience at any previous FIT test, irrespective of its timing, was predictive of behavior at future FIT rounds.

## Discussion

Most previous studies have examined participant CRC screening behavior over two [7, 8, 19] or three screening offers [11, 20, 21], with only one recent population-based, open-cohort study reporting [6] on four rounds. The current results concur with previous studies’ findings that younger age groups do not participate as readily in re-screening as older people do [6, 11]. Describing participatory patterns can better help to identify groups in the population at risk of not-participating at all or dropping out altogether from re-participation offers so that targeted interventions can be developed and implemented [8].

Although a strong and consistent finding in the screening literature is that male gender is a risk factor for non-compliance at first invitation [22–25], the results over multiple rounds suggest a possible cross-over or, at least, parity. In the UK [21], at the first invitation round, men were less likely to adhere to the second round compared to women (OR 0.72, *p* < .001) and men who did participate were less likely to adhere to the second round compared to women (OR 0.93, *p* < .05). However, by the third round the difference in consistent participation was non-significant (OR 0.96). These results are consistent with our previous finding that, over three annual rounds of screening, males were more likely to initially refuse but participate in later rounds (RR = 1.77, *p* < .001) [11]. This pattern has been sustained in the fourth round. There was no significant difference in participation between males and females (70% males, 72.5% females, (*χ2* (1) = .901, *p* = .343).

In the current study, as with previous research, participatory patterns were identified, and different demographic and behavioral variables were found to characterize consistent screeners, consistent non-screeners and inconsistent screeners [8, 11, 15]. People who consistently screened were older, especially women, and those with prior FIT participation. Results from the current study, similar to previous studies that have examined rescreening over two [8] or three [10, 11, 20] rounds, indicated that younger age (50–54 years), male gender and higher economic disadvantage emerged as the main risk factors for non-adherence to screening opportunities (OUT) or Changeable screening, (particularly to ‘drop out’).

Our results add to this previous research by examining screening patterns over four annual rounds, linking this to previous experience and demographics, and enabling the exploration of additional patterns within the ‘CHANGE’ category. Gender differences were found between those who screened intermittently (more likely to be female) and those who sustained their change in screening behavior (more likely to be male). Two additional patterns were identified within the Sustained Change category that differentiated between those who started screening and then discontinued (C_SIO_), and those who came into screening later and stayed (C_SOI_) with the latter being associated with having some prior (pre-trial) FIT experience, being in the workforce, of lower economic disadvantage and higher education. The ‘drop out’ participation pattern (C_SIO_) was observed in people who had not screened before the trial, had lower education levels and higher unemployment. The development of specific interventions targeted to subgroups such as these could be valuable, not only to encourage awareness and screening uptake in the first instance, but once screened, to encourage regular re-screening.

Prior dis-satisfaction with FIT testing was consistently associated with the likelihood (or not) of re-participating, similar to our previous results with this population [11, 15]. We previously found [11] that low self-efficacy (low confidence in one’s ability to do the screening activities) and low response efficacy (low confidence in the efficacy of the test itself) were associated with a non-participatory behavior pattern, a finding echoed by others [19, 26]. In addition, other researchers found that taking part in CRC rescreening would depend on not feeling too inconvenienced [27]. Results from the current study add to our previous findings that a previous negative experience, such as finding screening to be embarrassing or unpleasant, is associated with non-participation at any later rescreening round, even if that negative experience was well in the past. Other researchers have also demonstrated an association between negative prior experiences (embarrassment or disgust) and subsequent non-participation in re-screening for CRC [26] or breast cancer [28, 29].

The results can be viewed as consistent with the conclusions from a meta-analysis which established that past behaviour predicts substantial variance in subsequent behaviour [30]. Even though rescreening behaviour occurs only infrequently, that is annually or biannually, its regularity and social endorsement, and association with past compliance, suggest that intention to rescreen (and rescreening itself) are driven by cognitions that endorse the behaviour. Moreover, the significant negative association with previous dis-satisfaction further supports the largely planned, rather than habitual, nature of the behaviour. When developing interventions to educate people about CRC screening, and to take them through their first experience, care should be taken to make the experience as positive as possible to encourage screening maintenance.

Future interventions could include investigating the effect of different biological sample types, other than faeces, on screening participation and re-participation patterns. For example, in a recent Australian survey, participants overwhelmingly indicated a preference for a blood sample rather than the usual fecal sample for CRC screening [31]. In addition, future studies could examine the mode of screening kit delivery. In a French study [6, 20], ‘compliant’ participants were more likely to have received their kits from their health care provider, and ‘occasional’ participants were those more likely to receive them by post. The role of the healthcare provider in providing information about, and support for, CRC screening should not be underestimated [32]. In addition, if healthcare providers are made more aware of the demographic risk factors associated with lower screening uptake, they may be better equipped to identify patients at risk of not following through with screening recommendations, and who may benefit from additional support, education and encouragement.

This study was not without limitations. By the fourth round of screening offers, whether the more recent or less recent experience had the greater impact on the Round 4 screening behavior was difficult to test reliably due to low sample numbers. In the current study we did not test whether kit return time, test result or prior colonoscopy attendance influenced sustained participation in screening beyond three rounds, and these variables may be worth investigating in the future because there is evidence from a recent study that all three variables are associated with reduced uptake in re-screening [10]. These variables may have influenced participation over four rounds in the current study; we found Round 3 negative experiences were not significantly associated with Round 4 non-participation. In contrast, the results showed that those who were generally satisfied with their screening in Round 3 were less likely to participate in Round 4.

## Conclusions

The current study describes participation in four FIT screening rounds within a single cohort. This enables the development of a taxonomy of screening participation patterns including different forms of intermittency. Having greater information about factors associated with screening behavior provides the potential to identify demographic subgroups at risk and assist in the development of targeted interventions.

Subgroups likely to participate and re-participate in every rescreening offer are those who are older, female, not in the workforce and satisfied with a previous screening experience. People who start to participate in FIT screening and then change their participation and drop out (and sustain the change) are more likely to be male, younger, with lower education and no prior (pre-trial) FIT experience. In addition, the current study is the first to collect data about negative and positive FIT testing experiences prior to commencement of the program as well as at the end of each round of four rounds of offers. Negative prior experiences were found to be associated with future non-participation or a change in participation from ‘in to out’ up to the third, but not fourth, round of screening. Future interventions should investigate kit return time, test results and prior colonoscopy attendance as variables that may impact on the decision to rescreen, and focus on ensuring that the introduction into FIT screening is as positive an experience as possible to encourage maintenance in future screening activities.

## Abbreviations

CRC: Colorectal cancer
FIT: Fecal immunochemical testing
FOBT: Fecal-occult blood test
